# Advancing quantitative hazard banding using expanded probabilistic reference doses and high-throughput screening data for preliminary hazard assessment

**DOI:** 10.1016/j.namjnl.2025.100014

**Published:** 2025-03-05

**Authors:** Yu-Syuan Luo, Yu-Jia Yeh

**Affiliations:** a Institute of Food Safety and Health, College of Public Health, National Taiwan University, Taipei City, Taiwan; b Master of Public Health Program, College of Public Health, National Taiwan University, Taipei City, Taiwan

**Keywords:** Quantitative hazard banding, Probabilistic reference dose, Reproductive and developmental toxicity, Endocrine-active potential, High-throughput screening data, Globally harmonized system (GHS)

## Abstract

Data unavailability in health reference values poses a significant challenge in chemical risk assessment. Hazard banding (HB) offers a practical solution by categorizing chemicals into bands that represent increasing levels of health hazard. However, the strength and validity of these bands are often based on health-based guidance values from small sample sizes due to data scarcity. This study aims to develop quantitative hazard bandings using an expanded dataset of probabilistic reference dose values (pRfD) and endocrine-related quantitative high-throughput screening (qHTS) data. We hypothesize that quantitative hazard bandings can accurately reflect chemical hazards' nature and severity. pRfD values (*n* = 10,145) and oral equivalent doses (OEDs) from qHTS data (*n* = 3,520) were categorized into quintiles to derive five hazard bands (HB_pRfD_ and HB_qHTS_endo_). We evaluated the relationships between the assigned hazard bands and GHS health statements using heatmap and correlation analyses. Our findings suggest that HB_pRfD_ effectively captures the inherent severity of chemical hazards, while HB_qHTS_endo_ shows lower confidence in hazard characterization. Despite challenges with the discordance between animal toxicity data and qHTS data, integrating in vitro mechanistic evidence with animal data may improve predictive accuracy for higher-tier toxicity endpoints.

## Highlights

• A quantitative hazard banding framework is developed using recently expanded datasets.
• pRfD-based hazard bands effectively represent chemical hazard severity levels.
• Endocrine-based banding correlates with specific hazards but not general toxicity.
• Expanded pRfD and qHTS datasets improve hazard characterization for data-poor chemicals.

## Graphical abstract

**Figure:**
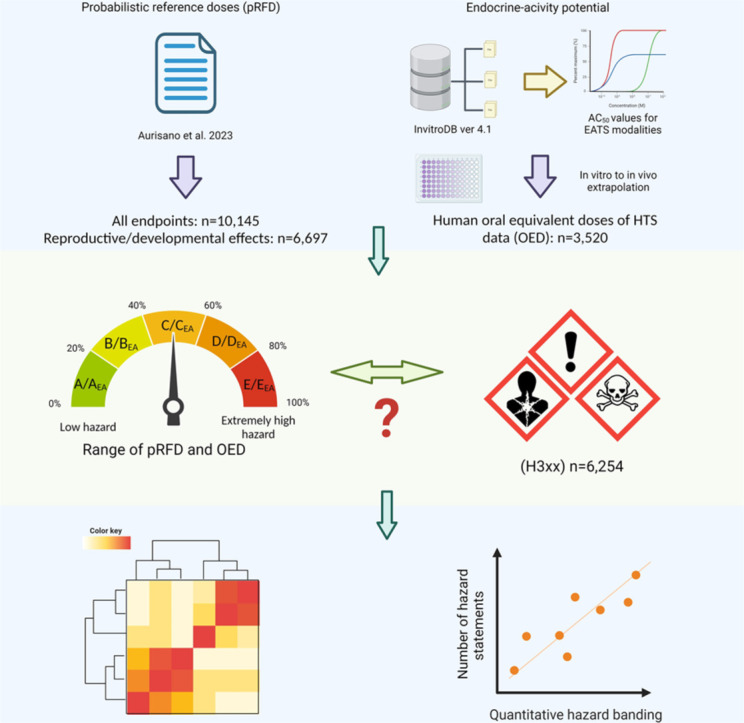

## 1 Introduction

Proper and unified classification and labeling are crucial to ensure effective hazard communication of chemicals. The globally harmonized system (GHS) of classification and labeling of chemicals has been widely adopted by the international community since its introduction in 2002 (Winder et al., 2005). The GHS hazard statements are designed to communicate the physical (**H2xx**), health (**H3xx**), and environmental (**H4xx**) hazards associated with chemicals (Ta, 2021). Specifically, GHS health hazard statements describe various health impacts posed by chemicals, including acute toxicity, skin irritation and corrosion, eye irritation and serious eye damage, skin or respiratory sensitization, target organ systemic toxicity, genotoxicity and germ cell toxicity, reproductive toxicity, and carcinogenicity (Winder et al., 2005). Each hazard class is further categorized to reflect the severity of the hazard. For instance, with respect to acute toxicity, H300 represents the most severe hazard (i.e., fatal if swallowed), while H305 indicates a less severe hazard (i.e., may be harmful if swallowed). The European Union (EU) regulatory framework, in addition to GHS, mandates the use of supplemental hazard statements to address specific properties of concern, such as endocrine-disrupting potentials to human health (EUH 380/381) and the environment (EUH 430/431) (ECHA, 2023). These health statements are included in Safety Data Sheets to effectively communicate both the nature and severity of hazards, which is particularly crucial for ensuring occupational health and safety.

Hazard banding (HB) categorizes chemical substances into bands of increasing health hazard (e.g., A, B, C, D, E) based on their GHS and EUH hazard statements, with Band E representing the most severe hazard and Band A the least severe (Scheffers et al., 2016). HB plays a crucial role in occupational assessment tools such as control banding (Zalk and Nelson, 2008) and risk prioritization (Marquart et al., 2008). HB provides a practical alternative for managing chemicals that lack occupational exposure limit (OEL) values. Many chemical producers and regulatory authorities have developed their own HB engines to assess data-poor substances; however, the strength and validity of these HB engines are often not rigorously evaluated. Scheffers et al. systematically investigated the strength and validity of four HB engines using the OEL values of 229 substances, finding that the Solvay occupational exposure banding (s-OEB) tool outperformed the others (Scheffers et al., 2016). This discrepancy is primarily due to the different allocation approaches used by each HB engine. The number of a chemical's hazard statements and the quantitative relationships between these statements and their inherent severities are therefore critical in determining the overall HB classification of a chemical.

The inherent severity of a chemical's hazard to human health can be quantified through health reference values, such as OELs and reference doses (RfDs). Generally, lower values of these reference points indicate a higher severity of the associated hazard. However, the regulatory values are available for only a small fraction of the chemical substances used globally, as the process of deriving these values is data-intensive, time-consuming, and requires substantial resources (Jolliet et al., 2021; Wignall et al., 2018). For instance, as of August 2024, the CompTox Chemicals Dashboard has collected information for 1218,248 chemicals, yet only 572 substances have been reviewed by the Integrated Risk Information System (IRIS) at the U.S. Environmental Protection Agency (US EPA) (USEPA, 2024a, 2024b). Recent advancements in computational toxicology, including the expansion of probabilistic reference dose/concentration values (Aurisano et al., 2024, 2023) and the high-throughput screening program Tox21 (Richard et al., 2021), offer promising tools for elucidating the relationships between the health hazard statements and quantitative toxicity information.

Using curated toxicity data from the U.S. EPA's Toxicity Value Database (ToxValDB) and the international Uniform Chemical Information Database (IUCLID), Aurisano et al. developed a semi-automated workflow to determine surrogate point of departure (POD) and corresponding health reference values in cases where regulatory assessments are unavailable. The authors derived probabilistic reference doses (defined as a 1 % incidence at 95 % confidence) and human population effect doses (defined as a 10 % incidence) for 10,145 chemicals. This effort significantly expanded the coverage of health reference values from 744 to 8023 for general non-cancer effects and from 41 to 6697 for reproductive and developmental effects (Aurisano et al., 2023). The expanded dataset provides a valuable benchmark for evaluating health hazard statements.

Quantitative high-throughput screening (qHTS) data from the Toxicity Forecaster (ToxCast) and Tox21 programs provide comprehensive insights into the mechanisms of chemical toxicity. Such data has been utilized for prioritizing chemicals for toxicity testing or environmental monitoring, as well as for the validation and development of novel assays and toxicity prediction (Jeong et al., 2022). For example, the integration of qualitative and quantitative data from nuclear receptor (NR)-related assays is extensively used to characterize the endocrine-active potential of chemicals (Luo et al., 2023; Luo and Wu, 2021) and to develop prediction models (Stanojevic et al., 2023; Wang et al., 2021). The publicly available qHTS dataset, InvitroDB, encompasses NR-related assays for approximately 10,000 test substances (Richard et al., 2021). This extensive knowledge of endocrine-active potential can be leveraged to reassess existing health hazard statements and determine the necessity for supplemental hazard statements regarding endocrine-disrupting potentials.

We hypothesize that quintile assignments in quantitative hazard banding, utilizing recent expansions of pRfDs and qHTS data, can accurately reflect the overall hazard posed by chemical substances. This study evaluates the robustness and validity of the quantitative hazard banding framework through a comparative analysis of pRfD data, qHTS data, and existing GHS health hazard statements.

## 2 Materials and methods

### 2.1 Study design

**Fig. 1:**
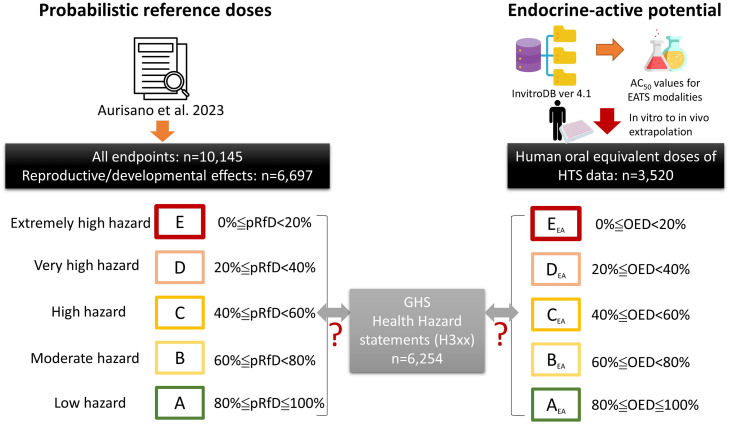
The overall workflow of this study. The quantitative hazard bandings were derived based on the 5 quintiles of the probabilistic reference doses (pRfD) curated from Aurisano et al. or the oral equivalent doses (OEDs) of the endocrine-related HTS data (minimum AC50 values). Next, the assigned quantitative hazard bandings were compared with the GHS health hazard statements.

In this study, we aimed to develop a classification framework for quantitative hazard banding and evaluate its strength and validity by comparing with GHS health hazard information (Fig. 1). This assessment was conducted using either an expanded dataset of health reference values for the general toxicity (Section 2.2) or endocrine-related HTS data (Section 2.3). Quantitative hazard bandings were derived based on the five quintiles of pRfDs curated from Aurisano et al., or the oral equivalent doses (OEDs) derived from endocrine-related HTS data (minimum AC_50_ values). Subsequently, we compared these assigned quantitative hazard bandings with the GHS health hazard information.

### 2.2 Probabilistic reference dose (pRfD)

The pRfDs for general noncancer effects and reproductive/developmental endpoints were obtained from Table S5 of Aurisano et al. (Aurisano et al., 2023). The source data file provides detailed information on the derived POD values, the number of underlying effect values, pRfD estimates, human population effect doses (HD_M_^10%^), and associated uncertainties for both general noncaner effects and reproductive/developmental effects. The pRfD is defined as the lower 95 % confidence bound of HD_M_^1%^, representing the estimated daily human dose at which 1 % of the population exhibits a level of effect (M) (Chiu et al., 2018). This calculation incorporates probabilistic uncertainty factors accounting for inter-species and inter-individual variability. Data on the toxicity of 10,145 test substances were gathered, comprising information on general noncancer effects for 8023 substances and reproductive/developmental effects for 6697 substances. Chemical Abstracts Service (CAS) identifiers were used to match chemicals across datasets (i.e., 2.3, 2.5), and only substances with CAS identifiers were included in the subsequent analysis.

### 2.3 In vitro quantitative high-throughput screening (qHTS) data

Chemicals with CAS identifiers curated in Section 2.2 served as the input chemical list for acquiring in vitro bioactivity values of qHTS data for *in vitro*-to-*in vivo* extrapolation (IVIVE). The qHTS assays were selected based on the classification listed in the Integrated Chemical Environment (ICE) (“Endocrine MOAs”, https://ice.ntp.niehs.nih.gov/Tools?tool=ivive, accessed date: 2024/01/12), representing the comprehensive endocrine-related processes (*n* = 171, Supplemental Table 1). All qHTS data available in ICE were retrieved from InvitroDB v3.5 (August 2022) and analyzed using the ToxCast Pipeline (tcpl, version 2.0.2) processing algorithm. The selected assays primarily target the nuclear receptors, including Estrogen (*n* = 56), Androgen (*n* = 36), Thyroid (*n* = 32), and other Steroid (*n* = 21) hormones (EATS) module, and other nuclear receptors (non-EATS module, *n* = 26). We converted in vitro AC_50_ values into OEDs using the IVIVE module in the Integrated Chemical Environment. The IVIVE parameters were set as follows: species = “Human”; body weight = “70 kg”(EFSA, 2012); ADME source = “Default”; model = “1C”. We successfully derived OED values for 3520 chemicals, where the calculated minimum OED for each test substance was used for deriving endocrine-active hazard bandings and correlation analyses.

### 2.4 Quantitative hazard bandings based on pRfD or endocrine-related HTS data

We derived the quantitative hazard bandings using general toxicity information (i.e., pRfD, *n* = 10,145, denoted as HB_pRfD_) and in vitro POD from endocrine-related qHTS data (i.e., minimum OED of endocrine-related HTS data, *n* = 3520, denoted as HB_qHTS_endo_), which represent the endocrine-active potential associated with the EATS module. The toxicity or POD values were divided into quintiles, forming the 5 categories (i.e., Q1, Q2, Q3, Q4, and Q5). The commonly-used five hazard bands were assigned to each category: ***E*** = “extremely high hazard”, Q1; ***D*** = “very high hazard”, Q2; **C** = “high hazard”, Q3; ***B*** = “moderate hazard”, Q4; and ***A*** = “low hazard”, Q5 (Table 1).

**Table 1:** Dose ranges for assigning hazard bandings based on pRfD or OED of qHTS data.

Hazard bandings	HB_pRfD_ (mg/kg/day)	HB_qHTS_endo_ (mg/kg/day)
E: Extremely high hazard	pRfD < 0.0196	OED < 2.469
D: Very high hazard	0.0196 ≦ pRfD < 0.078	2.469 ≦ OED < 16.586
C: High hazard	0.078 ≦ pRfD< 0.207	16.586 ≦ OED< 68.195
B: Moderate hazard	0.207 ≦ RfD<0.485	68.195 ≦ OED<196.141
A: Low hazard	0.485 ≦ pRfD< 28.948	196.141 ≦ OED< 21,187.686

### 2.5 GHS health hazard statements

The GHS health-related information was retrieved from the GESTIS substance database (IFA, 2024), including acute oral toxicity (H300-H304), dermal toxicity (H310-H317), ocular toxicity (H318 and H319), acute inhalation toxicity (H330-H335), narcotic effects (H336), mutagenicity or carcinogenicity (H340-H351), reproductive/developmental toxicity (H360-H362), and organ-specific or repeated dose toxicity (H370-H373). In total, we obtained the GHS health hazard statements for 6254 substances, with the data availability for each H statement summarized in Table 2.

**Table 2:** Data availability for each H statement in the GESTIS substance database.

H-code	Hazard Statements	Number of chemicals
H300	Fatal if swallowed	365
H301	Toxic if swallowed	816
H302	Harmful if swallowed	1904
H304	May be fatal if swallowed and enters airways	2605
H310	Fatal in contact with skin	253
H311	Toxic in contact with skin	511
H312	Harmful in contact with skin	447
H314	Causes severe skin burns and eye damage	1051
H315	Causes skin irritation	975
H317	May cause an allergic skin reaction	1606
H318	Causes serious eye damage	1437
H319	Causes serious eye irritation	1432
H330	Fatal if inhaled	525
H331	Toxic if inhaled	488
H332	Harmful if inhaled	562
H334	May cause allergy/ asthma symptoms/ breathing difficulties if inhaled	243
H335	May cause respiratory irritation	945
H336	May cause drowsiness or dizziness	137
H340	May cause genetic defects	67
H341	Suspected of causing genetic defects	272
H350	May cause cancer	310
H351	Suspected of causing cancer	307
H360	May damage fertility or the unborn child	372
H361	Suspected of damaging fertility or the unborn child	297
H362	May cause harm to breast-fed children	40
H370	Causes damage to organs	46
H371	May cause damage to organs	25
H372	Causes damage to organs through prolonged or repeated exposure	386
H373	May causes damage to organs through prolonged or repeated exposure	752

*Only substance with a CAS registry number was included in the analysis.

### 2.6 Data analysis and visualization

The curation and cleanup of the toxicity data was accomplished using package “dplyr” (version 1.1.2) in R (version 4.3.1, R Development Core Team) within RStudio (ver. 2023.06.0, build 421). Graphical representations, including bar graphs, scatterplots, and Spearman correlations (ρ), as well as the Chi-squared test for trends, were generated using GraphPad Prism 9 (version 9.5.0) (Fig. 3, Fig. 4, Fig. 5). Venn diagrams were produced via the R Shiny application “eulerr” (version 6.1.1, https://eulerr.co/).

## 3 Results

### 3.1 Revisit traditional toxicity data and new approach method (NAM) data for quantitative hazard bandings

**Fig. 2:**
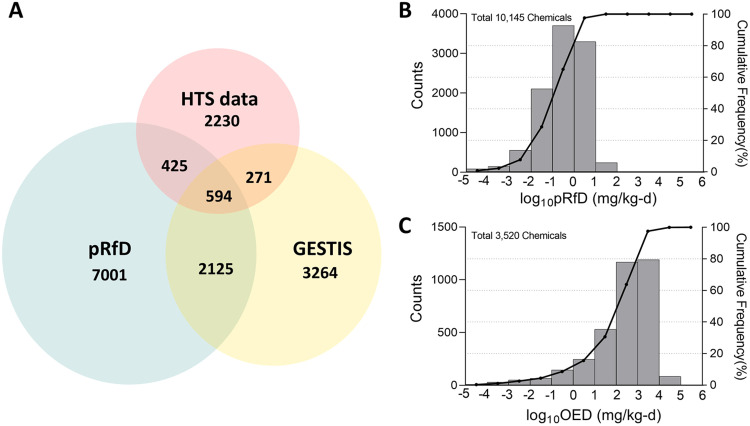
The data availability of probabilistic reference dose (pRfD), GHS health hazard information (GESTIS substance database), and oral equivalent dose (OED) of endocrine-related HTS data. (A) The Venn diagram displays the overlap in data availability among pRfD, GHS health hazard information, and OED data. The bar graph indicates the absolute frequencies (i.e., counts, left y axis) and the line graph shows the cumulative frequency (%, right y axis) of (B) pRfD values or (C) OED values.

The recent expansion of the regulatory toxicity database allows for pairwise comparisons among pRfD values, OED of endocrine-related qHTS data, and GHS health hazard information. The current analysis includes pRfD values for 7426 chemicals (with CAS identifiers), OED data for 3520 chemicals, and GHS health hazard information for 6254 chemicals. In total, 2719 chemicals have both pRfD and GHS health hazard information, 865 chemicals have both OED and GHS health hazard information, and 1019 chemicals have both pRfD and OED data (Fig. 2A). The curated pRfD values range from 3.3 × 10^−8^ mg/kg/day to 28.95 mg/kg/day, with most substances having pRfD values between 0.1 to 1 mg/kg/day (Fig. 2B). For the qHTS data, OED values range from 3.3 × 10^−6^ to 21,187.7 mg/kg/day, with most chemicals having OED values between 100 and 10,000 mg/kg/day (Fig. 2C).

### 3.2 Comparative analysis of pRfD-based quantitative hazard bandings and GHS health hazard information

**Fig. 3:**
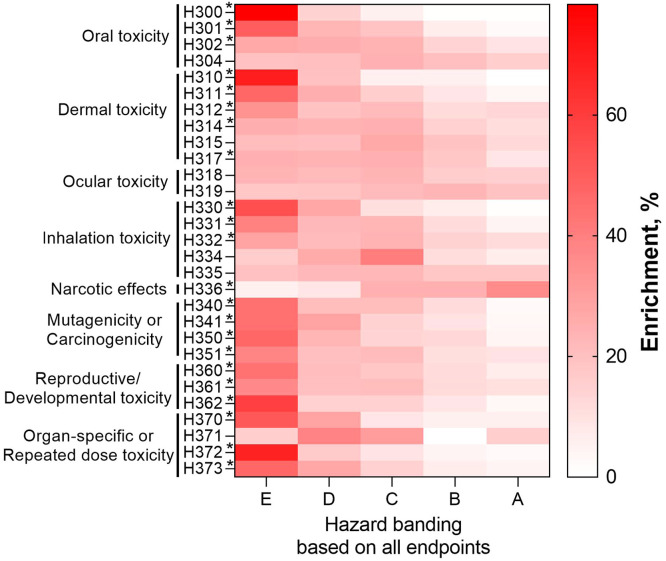
Comparative analysis of quantitative hazard bandings and GHS health hazard statements for 2719 substances. The heatmap visualizes the percent enrichment of hazard bandings corresponding to each GHS health hazard statements. An expected percent enrichment value of 20% is assumed for each hazard banding, with the sum of each row totaling 100%. The asterisk label denotes a statistically significant increasing/decreasing trend observed across hazard bandings (Chi-square test for trend, *p* < 0.05).

The assignment of HB_pRfD_ demonstrate strong agreement with the GHS hazard statements related to oral toxicity, dermal toxicity, inhalation toxicity, mutagenicity, reproductive/developmental toxicity, and organ-specific or repeated dose toxicity (Fig. 3). Across chemicals labeled with the same hazard statement, hazard banding E typically represents a higher percentage compared to hazard bandings A, B, C, and D, with an expected percentage of 20 % for each quintile. Furthermore, this trend is particularly pronounced for the hazard statements indicating more severe outcomes. For instance, the percentage of hazard banding E is higher for chemicals labeled with H300 (i.e., fatal if swallowed, 78.3 %) than those labeled with H301 (i.e., toxic if swallowed, 49.7 %), H302 (i.e., harmful if swallowed, 27.2 %), or H304 (i.e., may be fatal if swallowed and enters airways, 19.5 %). Similar patterns are observed among dermal (i.e., H310, fatal in contact with skin, 69.0 %; H311, toxic in contact with skin, 47.3 %; H312, harmful in contact with skin, 33.7 %) or inhalation toxicants (i.e., H330, fatal if inhaled, 54.2 %; H331, toxic if inhaled, 39.4 %; H332, harmful if inhaled, 28.6 %).

In contrast, the quantitative hazard banding classification struggles to differentiate the severity of ocular effects (i.e., H318, causes serious eye damage; H319, causes serious eye irritation), where each hazard banding showing similar percentage compared to the expected values (ranging from 15.2 to 23.5 % versus the expected 20 %). Interestingly, we observe a reverse relationship across quantitative hazard bandings for narcotic effect (i.e., H336, may cause drowsiness or dizziness). Most chemicals associated with narcotic effects are assigned with a quantitative hazard banding of A (36.2 %), following by B (24.6 %), C (24.6 %), D (8.7 %), and E (5.8 %).

**Fig. 4:**
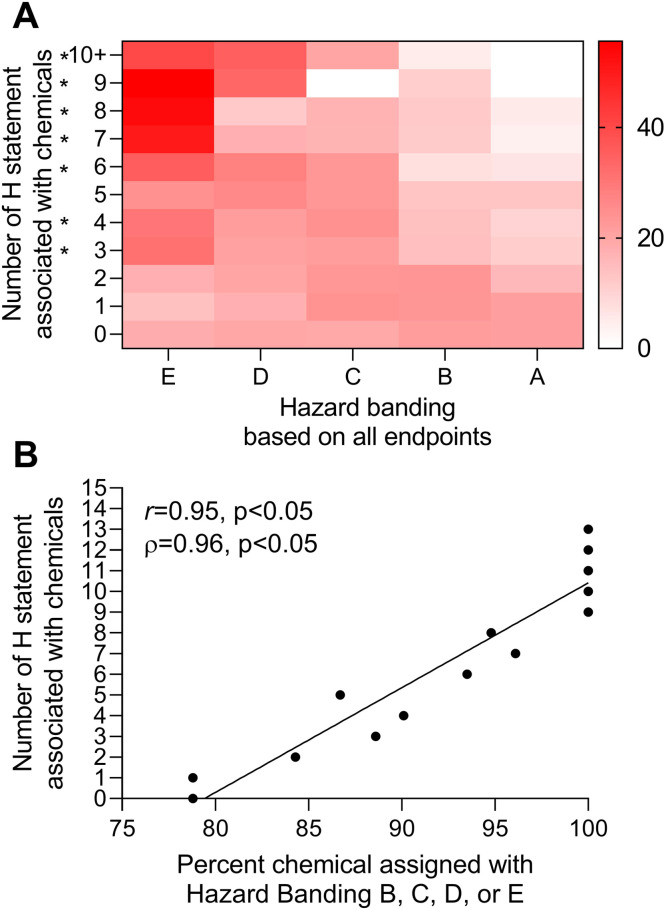
The relationship between quantitative hazard bandings and the number of H statements associated with chemicals. (A) The heatmap visualizes the percent enrichment of hazard bandings for each subgroup. The asterisk label denotes a statistically significant increasing/decreasing trend observed across hazard bandings (Chi-square test for trend, *p* < 0.05). (B) The scatter plot indicates a strong correlation between the number of H statements associated with chemicals and the percentage of chemical assigned with hazard banding B, C, D, or E. The x axis represents the summed percentage of chemicals assigned to hazard banding B, C, D, or E.

Next, we explored the correlation between the number of hazard statements associated with a given chemical and the pRfD-based quantitative hazard bandings (Fig. 4). Generally, chemicals linked to multiple hazard statements (*n* > 3) exhibit a higher percentage of quantitative hazard banding E compared to those associated with a single or no hazard statement (Fig. 4A). For chemicals associated with 7 or more hazard statements, over 50 % are assigned with the quantitative hazard banding of E (i.e., with 7 hazard statements= 50 %; 8 hazard statements= 53.4 %; 9 hazard statements= 55.6 %; 10 or more hazard statements, 40 %). Correlation analysis reveals a robust relationship between the number of hazard statements and the pRfD-based quantitative hazard banding linked to the same chemical (Fig. 4B, *r* = 0.95, *ρ*=0.96, *p* *<* 0.05*)*.

### 3.3 Comparative analysis of endocrine activity-based quantitative hazard bandings and GHS health hazard information

**Fig. 5:**
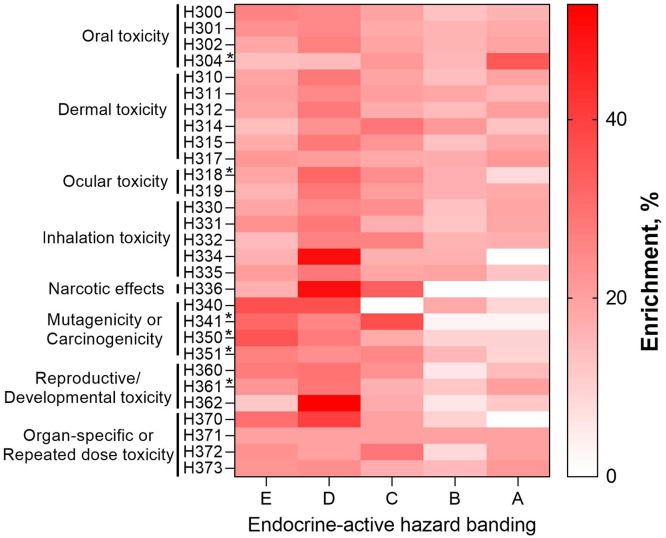
Comparative analysis of quantitative endocrine-active hazard bandings and GHS health hazard statements for 865 substances. The heatmap visualizes the percent enrichment of hazard bandings for each health hazard statements. The asterisk label denotes a statistically significant increasing/decreasing trend observed across hazard bandings (Chi-square test for trend, *p* < 0.05).

The assigned HB_qHTS_endo_ are significantly concordant with the GHS hazard statements related to mutagenicity (i.e., **H341**: suspected of causing genetic defects), carcinogenicity (i.e., **H350**: may cause cancer; **H351**: suspected of causing cancer), and reproductive/developmental toxicity (i.e., **H361**: suspected of damaging fertility or the unborn child, Fig. 5). Interestingly, we observe a significant yet opposite trend across quantitative hazard bandings for chemicals with hazard statement H304 (i.e., may be fatal if swallowed and enters airways). However, for the other endpoints examined, the endocrine-activity based quantitative hazard banding reveals no statistically significant trend.

### 3.4 Correlation analysis between the OEDs of endocrine-related HTS data and the reproductive/developmental point of departure (POD) values

**Fig. 6:**
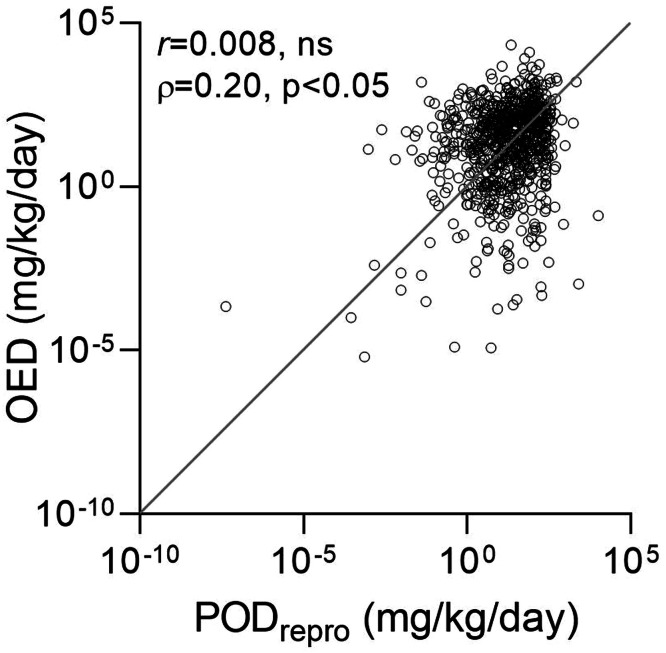
The correlation analysis between the oral equivalent doses (OEDs) of endocrine-related HTS data and the reproductive/developmental point of departure values.

The relationship between the OEDs of endocrine-related HTS data and the reproductive/developmental point of departure (POD_repro_) values was examined using Pearson or Spearman correlation analyses (Fig. 6, *n* = 793). A statistically significant, yet low degree of correlation was reported (ρ=0.2).

## 4 Discussion

The availability of health-based guidance values presents a significant challenge in health risk assessments, prompting the development of various approaches to generate data for hazard characterization. Among these, hazard banding requires the least amount of data compared to prescriptive process-based levels, provisional OELs, traditional OELs, and quantitative health-based OELs (Deveau et al., 2015). This makes hazard banding a practical alternative to regulatory OELs for hazard identification in occupational risk assessment. Additionally, semi-quantitative methods, such as the Threshold of Toxicological Concern (TTC), have been employed to screen and prioritize the risk assessment of chemical substances in cases where chemical-specific toxicity data are limited and human oral exposure is expected to be relatively low (More et al., 2019). The TTC specifies the human exposure levels that pose minimal toxicological concern, originally based on the distribution of the “no-effect” levels observed in 220 compounds from chronic studies (Frawley, 1967). The TTC approach has been evolving (Schüürmann et al., 2016; Stanard et al., 2015; Van Ravenzwaay et al., 2011) and is widely utilized in the safety assessments of food components (Kroes et al., 2004; Rennen et al., 2011; Renwick, 2004), fragrance materials (Patel et al., 2020), cosmetic ingredients (Kroes et al., 2007), and impurities in pesticide and pharmaceuticals (Dolan et al., 2005; Ghorab et al., 2024).

In this study, we hypothesize that the HB_pRfD_ or HB_qHTS_endo_ can serve as an alternative approach for hazard characterization. Our results show that HB_pRfD_ can better represent the nature and severity of a chemical's hazard. Herein, we derive the quantitative cut-off of pRfD for the initial hazard screening. Through a comprehensive comparison of pRfD data, qHTS data, and existing GHS health hazard statements, this research uncovers several noteworthy findings regarding the application of quantitative hazard banding utilizing pRfD or qHTS data in hazard characterization.

In general, the majority of the assessed chemicals exhibit pRfD values that are lower than the OEDs derived from endocrine-related qHTS data (i.e., 95 %), even when accounting for the common uncertainty factors of 100 for the OEDs (i.e., 73 %). This observation aligns with our previous analyses using a smaller dataset (*n* = 294), where 88 % of the evaluated animal drugs reported lower acceptable daily intake (ADI) values compared to the minimal OED derived from endocrine-related assays (Luo et al., 2023). However, several recent studies have concluded that in vitro qHTS data generally yield more protective PODs compared to in vivo PODs (Lu et al., 2024; Nyffeler et al., 2020; Paul Friedman et al., 2020), indicating that endocrine-related molecular events are typically not the most sensitive endpoints for chemical-induced in vitro effects. Additional considerations, such as the quantitative uncertainties associated with curve-fitting high-throughput bioactivity data, inter-individual variability, in vitro non-specific binding, extrahepatic metabolism and active transport, and the IVIVE may be necessary for the application of qHTS-based OEDs in chemical prioritization and health risk assessment (Pham et al., 2019).

Overall, the HB_pRfD_ effectively captures the severity of a chemical's hazard. The assigned HB_pRfD_ demonstrates strong concordance with the GHS hazard statements, with a notably higher enrichment of Band E observed in chemicals associated with more severe hazard codes such as H300 (“Fatal if swallowed”, 78.3 %) in comparison to less severe hazard codes like H301(“Toxic if swallowed”, 49.7 %) and H302 (“Harmful if swallowed”, 27.2 %). Similar patterns are observed in hazard codes related to dermal toxicity and inhalation toxicity. Furthermore, hazard codes such as H300, H310, H330, H340, and H350 demonstrate a high enrichment in Band E, which is used to categorize chemicals of highest concern by established hazard banding systems, including those developed by the German DGUV-IFA-Spaltenmodell and the UK HSE-Control of Substances Hazardous to Health (Scheffers et al., 2016). Additionally, chemicals associated with multiple hazard codes show enrichments of HB_pRfD_ Bands B, C, D, or E significantly exceeding the expected value (i.e., 20 %). These findings suggest that HB_pRfD_ classifications effectively communicate the inherent severity of chemical hazards.

Nevertheless, HB_qHTS_endo_ classification predominantly captures hazard-specific information. Statistically significant associations are observed between HB_qHTS_endo_ classifications and hazard codes related to carcinogenicity (i.e., H350 and H351) and reproductive toxicity (i.e., H361), but not with general toxicities. It is not surprising, as the receptor-mediated effects represent one of the key characteristics of carcinogens (Smith et al., 2016), male reproductive toxicants (Arzuaga et al., 2019), and female reproductive toxicants (Luderer et al., 2019). Transcriptional activation through nuclear receptors, such as the aryl hydrocarbon receptor, estrogen receptor, androgen receptor, and thyroid receptor, has been identified as a molecular initiating events in carcinogenesis (Chong et al., 2023; Safe and Zhang, 2022) and in the development of reproductive and developmental toxicities (Amir et al., 2021; Brown et al., 2023). In contrast, general toxicities may involve multiple modes of action, in which nuclear receptor-mediated activation plays a less central role.

Although endocrine-related in vitro PODs represent important molecular initiating events in the development of reproductive toxicity, their concordance with in vivo reproductive and developmental PODs is weak (ρ=0.2). This finding is consistent with previous analyses, which have generally shown a low correlation between in vitro and in vivo PODs (R^2^ ≤ 0.12) (Paul Friedman et al., 2020). However, this *in vitro*-to-*in vivo* discrepancy has been demonstrated to improve when utilizing Bayesian benchmark dose modeling for in vivo PODs, applying allometric scaling to account for interspecies differences, and incorporating human-relevant in vitro assays (Lu et al., 2024). Additionally, the presence of multiple key events in the progression towards in vivo apical phenotypes (i.e., adverse outcome) may further contribute to the observed discordance between in vitro and in vivo data.

This study is subject to several limitations. First, the analysis primarily relies on oral exposure data. Given the low correlation between pRfD and pRfC values (Spearman r = 0.19)(Aurisano et al., 2024, 2023), quantitative hazard bandings should be applied in a route-specific context. Second, pRfD values derived from surrogate POD may inherently involve a higher degree of uncertainty. Nonetheless, our findings demonstrate a strong concordance between the GHS hazard statements and pRfD values, indicating that surrogate PODs effectively capture the nature and severity of a chemical's hazard. Third, uncertainty associated with the use of NAMs data remains a critical challenge in advancing toward the animal-free “Next Generation Risk Assessment” (NGRA), particularly for higher-tier toxicity endpoints such as carcinogenicity and reproductive and developmental toxicity (Schmeisser et al., 2023). Additionally, the use of AC_50_ values instead of BMCL_10_ for deriving OED values could result in systematically higher OED values compared to most pRfD values. Despite these limitations, having a reasonable preliminary estimate, whether based on surrogate PODs or NAMs data, is preferable to having no data at all.

In conclusion, this study underscores the utility of quantitative hazard banding based on the expanded pRfD dataset. The established dose cut-offs for the five HB_pRfD_ classes can serve as a valuable tool for the preliminary hazard evaluation of new chemicals. Although the *in vitro*-to-*in vivo* concordance between animal toxicity data and NAM-based data poses a significant challenge for its application in NGRA, the integration of in vitro mechanistic evidence with animal data holds promise for improving the predictive accuracy of higher-tier toxicity endpoints.

## Declaration of generative AI and AI-assisted technologies in the writing process

During the preparation of this work, the author utilized ChatGPT-4 to enhance the quality of the English writing. Following the use of this tool, the author thoroughly reviewed and revised the content as necessary and assumes full responsibility for the content of the published article.

## CRediT authorship contribution statement

**Yu-Syuan Luo:** Writing – original draft, Visualization, Supervision, Resources, Project administration, Methodology, Investigation, Funding acquisition, Formal analysis, Data curation, Conceptualization. **Yu-Jia Yeh:** Writing – review & editing, Visualization.

## Declaration of competing interest

The authors declare that they have no known competing financial interests or personal relationships that could have appeared to influence the work reported in this paper.

## Data Availability

Data will be made available on request.

## Appendix Supplementary materials

Image, application 1
